# Liver Tuberculosis Presenting As Fever of Unknown Origin: A Case Report and Imaging Spectrum With a Review of Literature

**DOI:** 10.7759/cureus.47889

**Published:** 2023-10-28

**Authors:** Suchita Bahurupe, Sandip Dhote, Suresh Phatak, Kajal Mitra, Prashant Onkar

**Affiliations:** 1 Radiodiagnosis, NKP Salve Institute of Medical Sciences, Lata Mangeshkar Hospital, Nagpur, IND

**Keywords:** target ring sign, weight loss, tuberculoma, tuberculosis, hepatic tuberculosis

## Abstract

Hepatic tuberculosis is not commonly seen, and it can be easily missed unless there is strong suspicion. It presents clinically with non-specific symptoms like pain in the upper right abdomen, tenderness, mild fever, sweating at night, losing weight, feeling tired, and a lump in the abdomen. We are reporting a case of a 41-year-old female who presented with an intermittent history of fever and right hypochondriac pain for 10 years. Ultrasound and contrast-enhanced computed tomography (CECT) scans of the patient revealed a mass lesion with classical imaging findings of tuberculosis. Subsequently, a biopsy was conducted, confirming the presence of liver tuberculosis.

## Introduction

Hepatic primary nodular tuberculosis (TB), first documented by Bristowe in 1858, constitutes an uncommon manifestation of mycobacterial ailment as unexplained fever. This condition is characterized by TB affecting the liver and mild involvement of other bodily systems [1].

Tuberculosis bacilli can enter the liver through hematogenous dissemination, often from the lungs, or through local spread from the gastrointestinal tract. Miliary hepatic TB involves bacilli reaching the liver through the hepatic artery, leading to widespread tubercle distribution within hepatic lobules. On the other hand, localized hepatic TB primarily spreads through the portal vein from a gastrointestinal focus, resulting in larger tubercles near the portal triad region. Both miliary and localized hepatic TB are characterized by the formation of granulomas, which occur due to a cell-mediated immune response to TB antigens. It comprises macrophage aggregates, including Kupffer cells, along with surrounding lymphocytes and fibroblasts [2].

Hepatobiliary TB primarily impacts individuals within the age range of 11 to 50 years, with the highest occurrence of the condition noted during the second decade of life. The disease displays a higher frequency in males, with a ratio of 2:1. Nonetheless, isolated hepatic TB is more frequently observed during the fourth to sixth decade of life. It remains silent and is detected incidentally during evaluation for non-specific symptoms [3].

Various experts have discussed how hepatic TB appears in images produced by CT scans and MRI scans. The imaging finding of hepatic tuberculosis is not very specific, therefore, we often need to do further tests, such as histopathologic examinations, to confirm the diagnosis accurately [4].

## Case presentation

A female of age 41 came for assessment as she had had a fever (100 to 103 degrees Celsius) for nearly six weeks. Before admission, she had undergone a one-week course of antibiotics without displaying any clinical improvement. The medical history indicated occasional nocturnal hyperhidrosis (night sweats) and loss of weight. For 10 years, she has been experiencing these symptoms, during which she sought advice from various local medical hospitals. While the symptoms would improve temporarily following treatment, they would recur after a few months, prompting her to present with the same complaints once again. The absence of respiratory symptoms and signs was noted. A physical examination unveiled a slender physique. Hepatomegaly and splenomegaly were evident, while there were no findings of ascites, arthralgia, or cutaneous manifestations. Laboratory tests suggested a normal value, except for increased alkaline phosphate values and slightly increased lymphocyte counts. Tests for other pathogens like malaria, typhoid, and dengue also revealed negative results.

**Figure 1:**
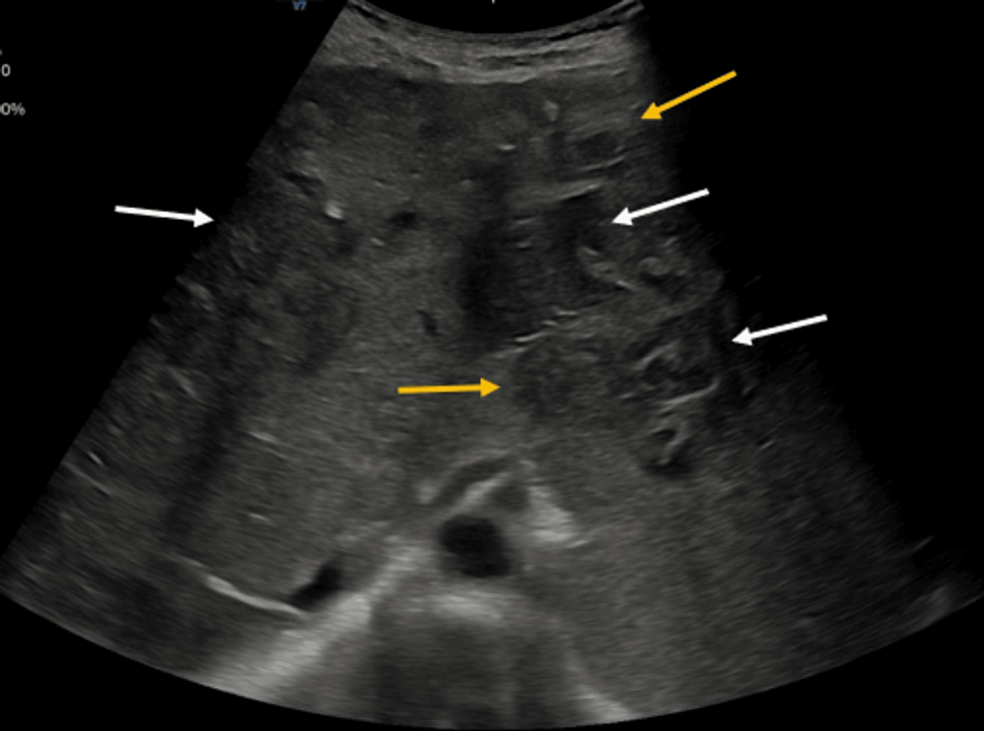
On ultrasound, multiple hypoechoic lesions are noted scattered throughout the liver parenchyma (white arrows). Some lesions show a target ring appearance that is a central hypoechoic area surrounded by hyperechoic rims (yellow arrows).

Ultrasound examination unveiled the presence of numerous, well-defined hypoechoic, solid-appearing, circular regions of average size from 1 to 2 cm. These areas were diffusely distributed throughout the hepatic parenchyma (Figure 1).

**Figure 2:**
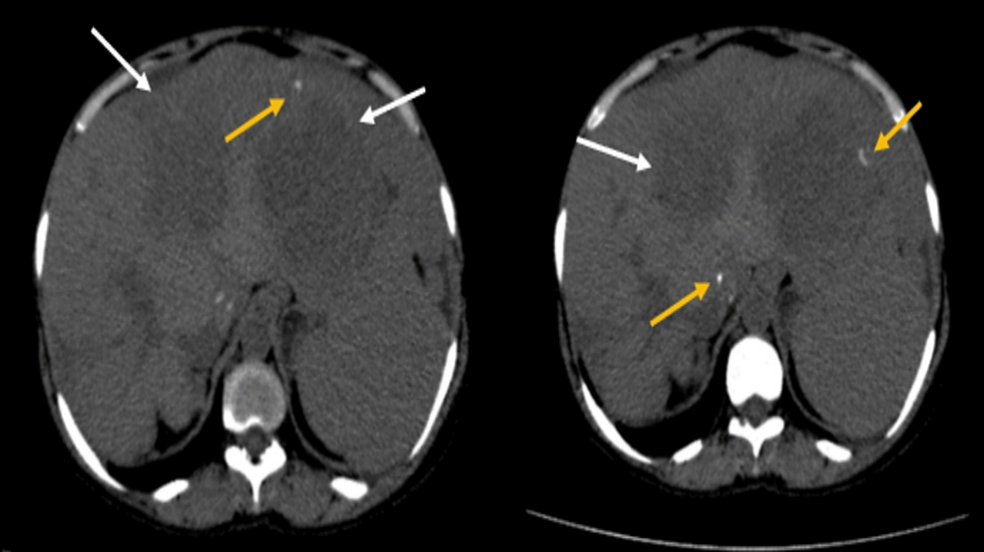
On computed tomography, multiple hypodense lesions are noted scattered throughout the liver parenchyma (white arrow). A few calcific foci are noted (yellow arrow).

Plain abdominal CT imaging revealed hepatomegaly with multiple discrete round lesions of average size 1.7 x 1 cm noted within the liver, characterized by low-density attenuation (Figure 2).

**Figure 3:**
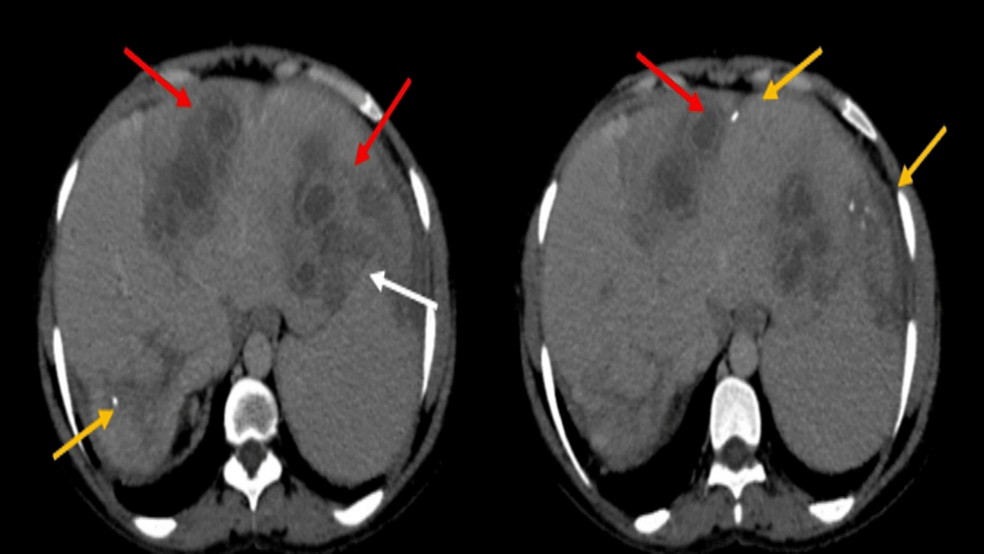
On contrast-enhanced CT, hypodense lesions are noted. Some give rim enhancement (target appearance) (red arrows), while others are not enhanced (white arrows). A few calcific foci are noted (yellow arrow).

Following intravenous contrast administration, the hypoattenuated foci exhibited discernible contrast enhancement at their periphery. Some lesions showed calcification within (Figure 3).

**Figure 4:**
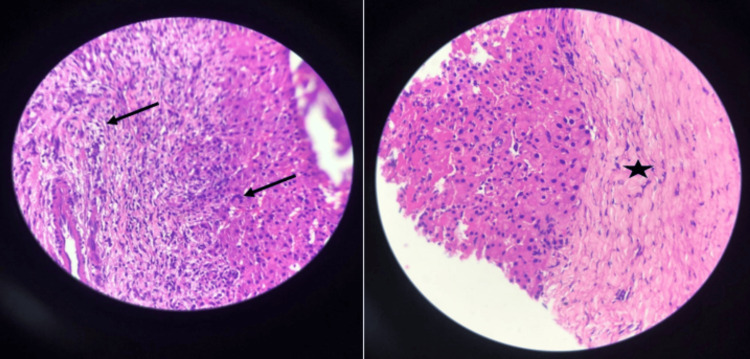
Core biopsy histopathological examination of the hepatic lesion reveals giant cell granuloma (black arrows) and extensive caseous necrosis (black asterisks).

Based on the findings we observed in the CT scans, our initial assessment suggested the infectious processes leading to the formation of granulomatous lesions; alternatively, the presence of spread-out metastatic growths that might have originated from a concealed primary tumor could also be attributed. To rule out a differential diagnosis, the patient underwent a liver biopsy, which suggested granuloma with central caseous necrosis and scattered acid-fast bacilli (Figure 4).

 The patient was started on anti-tubercular treatment(ATT) for six months and antipyretics. 

## Discussion

Fever of uncertain origin is a prevalent clinical challenge. Tuberculosis is a significant contributor to both mortality and illness on a global scale, particularly in tropical regions. In cases of TB affecting the liver, it is often associated with widespread infection, leading to a diffuse pattern of involvement where the liver tissue displays numerous tiny nodules, resembling millet seeds. In contrast, isolated occurrences of TB solely in the liver are infrequent in clinical practice, with only a handful of isolated instances and brief collections of cases documented in existing medical literature [3].

Hepatic TB is categorized as follows: a) primary acute pulmonary TB with hepatic involvement; b) miliary TB; c) primary TB of the liver; d) tuberculoma (abscess); e) chronic pulmonary TB with hepatic involvement; and f) tuberculous cholangitis [1].

Primary TB of the liver, recognized by hepatic lesions exceeding 2 cm, is termed macronodular or pseudotumoral TB. Comparatively uncommon to the miliary type, it often appears as solitary or multiple hepatic masses of varying sizes. Distinguishing these from neoplastic or infectious lesions can be challenging. On ultrasound, these lesions present a range of features, from heterogeneous hypoechoic to mixed hypoechoic-hyperechoic with anechoic regions. On plain CT, their characteristics vary with the disease stage. On plain CT, the granulomatous lesions show calcification in chronic cases, which differentiates them from metastatic lesions. Non-caseating granulomas show hypodensity with minimal enhancement, resembling other conditions. Lesions with caseous necrosis appear differently based on internal liquefaction. Sometimes tubercular liver abscesses can rupture, causing complications like perihepatic abscesses or infective peritonitis [3].

Miliary TB is a common form of liver involvement by TB, often linked with widespread disease. Lesions appear as small hypoechoic to isoechoic areas on sonography, occasionally hyperechoic. On CT, these microabscesses show low attenuation, nodular or cystic appearance, and minimal contrast enhancement. Distinguishing them from metastases, lymphoma, or granulomatous diseases can be challenging [3].

Magnetic resonance imaging of primary TB also revealed histological variations between the core and edges of tuberculoma in both T1-weighted images (T1WI) and T2WI. An MRI could distinguish caseation necrosis from peripheral granulomatous tissues; no need for contrast is required as compared to CT; however, MR offered limited additional insight here. Tuberculoma MR findings can be nonspecific and change during the disease course, similar to CT. While useful for large lesions, both CT and MR might miss smaller ones [5].

Tuberculous cholangitis, though uncommon and mostly seen in children, frequently leads to obstructive jaundice. Pathological findings indicate localized or widespread duct dilation with thickening and stiffening of the duct walls. Imaging displays irregularly dilated intrahepatic ducts or widespread tiny calcifications along the bile ducts, the latter being a distinctive trait of this condition. The serohepatic type, the rarest among the three types of hepatic TB, presents as miliary tuberculous lesions beneath the liver capsule or a "frosted liver" due to thickened subcapsular tissue. Imaging descriptions of this type are scarce [4].

The differential diagnosis of macronodular hepatic tuberculoma encompasses a range of hepatic masses due to its varied presentation, necessitating careful differentiation from malignant hepatic tumors. On CT imaging, its features often mimic those of necrotic tumors like metastatic carcinoma and hepatocellular carcinoma. The ringed appearance is a common trait shared by various hepatic lesions, while the central hyperintensity surrounded by a less hyperintense rim, resembling a "target" appearance, mirrors the presentation of hepatic metastases with liquefaction necrosis. Distinguishing characteristics include the association with substantial lesions exceeding several centimeters, a rarity in hepatic tuberculomas, and the presence of concurrent abdominal or mediastinal lymphadenopathy with distinct imaging features, coupled with hepatomegaly, potentially indicative of tuberculosis. However, a definitive diagnosis and appropriate management necessitate a liver biopsy [5].

## Conclusions

Hepatic TB presents in various aspects, and its visual representation can sometimes closely resemble other, more commonly seen primary or secondary liver lesions. Diagnosing isolated hepatic TB based solely on imaging can be particularly challenging due to its largely nonspecific features. Nevertheless, in regions with a high prevalence of the disease and suitable clinical contexts, unusual imaging of a liver lesion should raise suspicion for hepatic TB. While image-guided biopsy is typically needed for a definitive diagnosis, the presence of calcifications and simultaneous involvement of extrahepatic areas (such as the spleen, lungs, and lymph nodes) should raise consideration for hepatobiliary TB.
